# Translation and validation of a Turkish version of the Xerostomia Inventory XI in patients with primary Sjögren’s syndrome

**DOI:** 10.3906/sag-2005-157

**Published:** 2021-10-21

**Authors:** Bilge BAŞAKCI ÇALIK, Elif GÜR KABUL, Aylin KESKİN, Sinem BOZCUK, Hande ŞENOL, Veli ÇOBANKARA

**Affiliations:** 1 School of Physical Therapy and Rehabilitation, Pamukkale University, Denizli Turkey; 2 Department of Biostatistics, Faculty of Medicine, Pamukkale University, Denizli Turkey; 3 Department of Rheumatology, Faculty of Medicine, Pamukkale University, Denizli Turkey

**Keywords:** Sjögren’s syndrome, Xerostomia Inventory, validation

## Abstract

**Background/aim:**

The aim of this study was to assess the reliability and validity of Turkish version of the Xerostomia Inventory XI in patients with primary Sjögren’s syndrome (pSS).

**Materials and methods:**

A cross-sectional survey study design and analysis were used to assess the reliability and validity of the Xerostomia Inventory XI. A total of 69 patients with pSS (5 males, 64 females; mean age = 54.81 ± 8.77 years) were included. The Xerostomia Inventory XI (TR) was applied twice at an interval of 15 days. The test-retest reliability was assessed with the intraclass correlation coefficient (ICC), and the internal consistency of multiitem subscales by calculating Cronbach’s alpha values. The correlations between ESSPRI, basal and stimulated salivary flow (BSF-SSF), Oral Health Impact Profile-14 (OHIP-14) and Oral Health-Related Quality of Life-UK (OHRQoL-UK) Questionnaire were evaluated to determine the construct validity.

**Results:**

The ICC value for test/retest reliability of the Xerostomia Inventory XI (TR) was 0.993. The internal consistency was 0.869. There were low to high correlations between Xerostomia Inventory XI (TR) and ESSPRI, BSF, SSF, OHIR-14 total and OHRQoL-UK total.

**Conclusion:**

The Turkish version of the Xerostomia Inventory XI was found to be clinically valid and reliable to be used in clinical evaluations and rehabilitation interventions in patients with pSS.

## 1. Introduction

Primary Sjögren’s yndrome (pSS) is a chronic, multisystemic, autoimmune and idiopathic disease that involves all exocrine glands, mainly the salivary and tear glands. Lymphocytic infiltration of the salivary and tear glands causes complaints of xerophthalmia and xerostomia. In addition to this, there may be complaints such as dry nose, dry cough, or dry skin [1] . These dryness complaints greatly affect daily quality of life [2]. The pSS prevalence in Turkey has been found to be 0.72% according to the American-European classification criteria and to be 1.56% according to the European criteria [3].

SSaliva, which plays a critical role in oral health and maintaining comfort, has antibacterial, lubricant, remineralising, digestive, buffering and cleansing properties [4,5]. Dry mouth (xerostomia), which is one of the main symptoms in patients with pSS, reduces the quality of life. Patients complain about a reduced amount of saliva, which causes problems in speaking, swallowing and chewing of food, thereby significantly reducing quality of life. Patients feel the need to drink water, especially when they eat dry food, wake up, or talk for a long time [6]. The amount of saliva can be increased with the use of chewing gum, sugar-free desserts, lemon or artificial saliva products [7].

A decrease in the amount of saliva negatively affects the intraoral flora, and changes also occur in the oral flora of patients with pSS. The effect of the oral mucosa in these patients is to reduce Ig-A secretion and weaken the antibacterial defence system. It is extremely important that the pH of the oral cavity is continuously protected. A reduction in oral cavity pH is common in these patients, but if the pH can be kept stable, the impairment in mineralisation is reduced. A change in pH and loss of the antibacterial effect of saliva cause tooth decay. Therefore, patients should pay attention to oral hygiene [8]. 

- Patients with pSS are diagnosed according to the American-European Consensus Group  or the American College of Rheumatology (ACR)/European League Against Rheumatism (EULAR) classification criteria [9,10]. Although these criteria provide an advantage for diagnosis, xerostomia is not correlated with disease activity [11]. Xerostomia is a subjective feeling. Therefore, instead of a tool to evaluate this symptom with a single question, a measurement tool should be used which can evaluate it from multiple angles and includes the complexity of the symptom [3]. The relationship between the subjective data of dry mouth of pSS patients and saliva flow rate has been reported to be weak [12]. Therefore, it has been suggested that it would be more appropriate to evaluate the symptoms of xerostomia using scales such as isual nalogue cale, Xerostomia Inventory and Sicca Symptom Inventory [13]. Although the correlation is weak, saliva flow rate was used as objective data in this study as in other version studies, but in addition, questionnaries were applied to assess the validity of the Turkish version in the evaluation of oral symptoms. These questionnaires were the EULAR Sjögren yndrome atient VASSPeporting ndex (ESSPRI) and quality of life (Oral Health Impact Profile (OHIP-14) Oral Health-Related Quality of Life-United Kingdom (QHRQOL-UK). 

The Xerostomia Inventory XI (XI) is a widely-used questionnaire in disease-specific assessments and research. It examines the symptoms of xerostomia multidimensionally and consists of 11 items. The original English version of the survey was developed in 1999 by the Australian dentist, Murray Thompson et al [14]. The survey items show that both experimental and behavioral features have excellent reliability, validity, responsiveness [11]. XI enables patients to express their complaints clearly, as the questionnaire consists of yes/no binary answers, and multiple responses ranging from 1 (never) to 5 (RI) “”very often), thereby allowing patients to express the severity of symptoms without limitations.

“”The XI has been translated and culturally adapted in many countries such as Spain [13], Greece [15], South Korea [11], Portugal [14] among others [16,17]. No validity and reliability studies of the XI have been conducted previously in Turkish. The aim of this study was to assess the reliability and validity of the Turkish version of the XI in patients with pSS.

## 2. Materials and methods 

### 2.1. Participants

The study sample was formed of patients who presented at the heumatology linic. The study was completed with a total of 69 patients, considering that the number of samples should be at least 5 times the number of items in the questionnaire [16] and the incidence of the disease is low [18].RC

Inclusion criteria were a diagnosis of pSS according to the revised ACR/EULAR classification criteria [10], medical treatment stable for 6 months before the study, age 2065 years, ability to speak and understand Turkish fluently and voluntary participaton in the study. 

Exclusion criteria were defined as any treatment between the first test and the retest, having received radiation to the head and neck region, other causes of xerostomia, cancer patients, salivary gland inflammation, a history of another rheumatic disease, any psychiatric or cognitive impairment, acquired immunodeficiency syndrome, pre - existing lymphoma, graft versus disease, or a history of anticholinergic drug use. The data of patients for whom any changes in drug treatment were made during the study were not included and the participation of the patient was terminated.

### 2.2. Procedures

A cross-sectional survey study design was used to assess the reliability and validity of the Turkish version of XI.

### 2.3. Clinical data

Demographic data were collected in face-to-face interviews. All participants were evaluated under the same conditions by the same rheumatologist and physiotherapist experienced in the field of rheumatological rehabilitation. The xerostomia symptom of all the patients was evaluated with XI, disease activity with the ESSPRI, oral health with the OHIP-14 and oral health-related quality of life with the QHRQOL-UK scale. In addition, after a 5-min rest in a quiet laboratory environment, basal and stimulated (citric acid 2%) saliva flow rate was measured with the saliva flow rate test. The evaluations were made in a single session lasting 4560 min.

ute - utesFor the retest, the patients completed the XI for a second time after an interval of one week.

#### 2.3.1. Translation and cultural adaptation of XI

The recommended procedures of XI for Turkish validity and reliability were followed [19,20] and were carried out in 5 stages. First, the XI was translated from English into Turkish by 2 independent translators whose native language was Turkish and had a good level of English. A synthesis of the translations was produced. This was then back-translated by 2 other independent translators with English as a foreign language and good knowledge of both languages. No translator was able to access the original version and they were not informed about the concept of the survey. Later, all versions of the questionnaire were combined by 4 physiotherapists who had not been involved in the translation but were experienced in the treatment of individuals with pSS and by translators who made bidirectional translations. Then, the prefinal version was prepared and applied to 10 patients with pSS to evaluate each item. The final Turkish version of XI (XI-TR) was produced when there was no problem about the clarity and comprehensibility of the statements of each item (Table 1). 

**Table 1:** The original and Turkish version of the Xerostomia Inventory XI.

Items	Turkish Xerostomia Inventory-XI (TR)	Xerostomia inventory (XI)
1	Yiyecekleri yutmak için yanında sıvı tüketirim.	I sip liquids to aid in swallowing food.
2	Yemek yerken ağzımın kuruduğunu hissederim.	My mouth feels dry when eating a meal.
3	Geceleri su içmek için uyanırım.	I get up at night to drink.
4	Ağzımda kuruluk hissederim.	My mouth feels dry.
5	Kuru gıdaları yemekte zorluk çekerim.	I have difficulty in eating dry foods.
6	Ağız kuruluğunu azaltmak için şeker veya pastil kullanırım.	I suck sweets or cough lollies to relieve dry mouth.
7	Bazı yiyecekleri yutmakta zorluk çekerim.	I have difficulties swallowing certain foods.
8	Yüzümde kuruluk hissederim.	The skin of my face feels dry.
9	Gözlerimde kuruluk hissederim.	My eyes feel dry.
10	Dudaklarımda kuruluk hissederim.	My lips feel dry.
11	Burnumun içinde kuruluk hissederim.	The inside of my nose feels dry.
Score		
1	Hiçbir zaman	Never
2	Hemen hemen hiç	Hardly ever
3	Bazen	Occasionally
4	Oldukça sık	Fairly often
5	Çok sık	Very often

#### 2.3.2. Xerostomia Inventory XI (XI)

The XI consists of 11 items. The patients were asked to select the best response for each item describing their symptoms during the previous two weeks. The responses are scored between 1 and 51: ever, 2: , as :Nardly ever, 3: ccasionally, 4: airly often, and 5: ery often. The total of the item scores provides the total score ranging between 11 and 55, with a higher score indicating more severe symptoms [7].

#### 2.3.3. Oral Health Impact Profile-14 (OHIP-14)

The OHIP-14 is a scale used to evaluate oral health comprehensively, questioning physical and mental conditions. It consists of 14 items, evaluating 7 areas of functional limitations, physical pain, mental distress, physical disability, social disability, mental disability and disability. Responses are given with a 5-point Likerttype rating, with a total score ranging from 0 to 56. A high score indicates that the patient has more difficulties and a decreased quality of life. Turkish validity and reliability studies have been conducted for the OHIP-14 [21,22]. 

#### 2.3.4.Oral Health-Related Quality of Life-United Kingdom (OHRQoL-UK)

This consists of 16 items to evaluate how oral and dental health affect the general health and quality of life of individuals. It is divided into 4 main groups of physical state, symptom, social state and mental state. Items are scored with a 5-point Likert-type rating to give a total score of between 16 and 80. The responses are scored as 1 =very badly, 2= badly affected, 3= no effect, 4= good effects, and 5 = very good effects. A higher total score indicates a better quality of life [22,23]. 

#### 2.3.5. EULAR Sjögren yndrome atient SPeported ndex (ESSPRI)

**R** **I**ESSPRI scoring is a symptom severity assessment scale completed by the patient and used in the evaluation of SS. Items referring to patient complaints of fatigue, pain and dryness are scored from one to ten. The arithmetic average of the scores is then calculated to give a final value. ESSPRI score <5 is considered as an acceptable disease condition, and a score of ≥5 as a sign of high activity [24]. 

#### 2.3.6. Salivary flow rate test 

For this measurement, the patients with pSS were asked to attend the clinic at 09:00 after overnight fasting. To determine the amount of basal saliva, the individual was rested for 5 min before starting the measurement. Then, the tare of the measuring cup was determined and the individual was asked to collect his or her saliva into the measuring cup for 10 min in a quiet environment. The amount of saliva collected was measured on a sensitive scale and recorded in grams. The method for measuring the amount of stimulated saliva was the same following the application of two drops of citric acid (2%) onto the tongue to stimulate saliva production [25]. 

#### 2.3.7. Statistical analysis

All statistical analyses were performed using SPSS v. 25.0 software (IBM SPSS, Armonk, NY, USA).  Continuous variables were expressed as mean ± standard deviation (SD), median (minimum-maximum)values and categorical variables as number (n) and percentage (%). Construct validity was investigated using the Spearman rank correlation coefficient. Correlations were categorized as low (r:0.100.49), moderate (r:0.500.69) or high (r:0.701.00). The scale reliability was measured using the Cronbach’s alpha (a) coefficient.  n---The testretest method was used to assess the scale’s stability over time. The correlation between the two measurements was calculated using the intra class correlation coefficient (ICC). Internal consistency reliability was evaluated with Cronbach’s alpha coefficients. 

## 3.Results

The study group of pSS patients comprised 92.8% females with a mean age of 54.81 ± 8.77 years. The demographic and clinical characteristics of the patients included in the study are given in Table 2. 

**Table 2:** Demographic and clinical characteristic of participants.

		N	%
Sex	Female	64	92.8
	Male	5	7.2
		Mean ± SD	
Height (m)		158.99 ± 7.29	
Body weight (kg)		72.45 ± 13.62	
		Median (min-max)	
Age (years)		56 (26–65)	
Duration of disease (years)		6 (1–21)	
Chisholm & Mason grade		3 (1–4)	

The descriptive statistics of the XI-TR test and retest, ESSPRI, salivary flow rate test, OHIP-14 and OHRQoL-UK questionnaires are summarized in Table 3. Descriptive analyses of the XI-TR test/retest are given in Table 4.

**Table 3:** Descriptive statistics of applied scales and clinical findings.

	Mean ± SD
Xerostomia Inventory- test total score	36.41 ± 7.67
OHIP-14 total	24.54 ± 10.42
OHRQOL-UK total	39.52 ± 4.92
	Median (min-max)
Xerostomia Inventory- retest total score	38 (21–51)
ESSPRI	8 (3.3–10)
Basal amount of saliva (gr)	1.11 (0–5.74)
Stimulated amount of saliva (gr)	1.44 (0.1–8.26)
OHIP-14 functional limitation	4 (0–8)
OHIP-14 physical pain	3 (0–7)
OHIP-14 psychological discomfort	6 (0–8)
OHIP-14 physical disability	2 (0–7)
OHIP-14 psychological disability	3 (0–7)
OHIP-14 social handicap	4 (0–8)
OHIP-14 handicap	3 (0–8)
OHRQOL-UK symptom	5 (2–10)
OHRQOL-UK physical status	12 (7–15)
OHRQOL-UK psychological status	12 (7–15)
OHRQOL-UK social status	11 (7–12)

**Table 4:** Mean scores, standard deviations and test-retest reliability of the Xerostomia Inventory-XI (TR).

Items	Test	Retest	ICC	95% CI of ICC (lower– upper)
	Median (min-max)	Median (min-max)		
1	3 (1–5)	3 (1–5)	0.994	0.99–0.996
2	3 (1–5)	3 (1–5)	0.98	0.968–0.988
3	3 (1–5)	3 (1–5)	0.973	0.956–0.983
4	4 (2–5)	4 (2–5)	0.949	0.917–0.968
5	3 (1–5)	3 (1–5)	0.986	0.977–0.991
6	1 (1–5)	1 (1–5)	0.991	0.985–0.994
7	3 (1–5)	3 (1–5)	0.983	0.972–0.989
8	4 (1–5)	4 (1–5)	0.939	0.901–0.962
9	5 (2–5)	5 (3–5)	0.93	0.887–0.957
10	5 (3–5)	5 (3–5)	0.853	0.762–0.909
11	3 (1–5)	3 (1–5)	0.951	0.92–0.969
	Mean ± SD	Median (min-max)		
Total	36.41 ± 7.67	38 (21–51)	0.993	0.988–0.995

ICC: intraclass correlation coefficient, CI: confidence Interval.ICC values less than 0.50 indicate poor reliability, values between 0.50 and 0.75 indicate moderate reliability, values between 0.75 and 0.90 indicate good reliability, values greater than 0.90 indicate excellent reliability (Koo, 2016).

### 3.1. Reproducibility

In all items, the test-retest examinations of the XI-TR scale were found to have a very high ICC. The obtained ICCs and confidence intervals are given in Table 4. The scale items werre detemined to be reliable.

### 3.2. Internal consistency reliability

The internal consistency coefficient (Cronbach’s lpha value) of the XI-TR scale was found to be 0.869 indicating that the scale was quite reliable. When the “Cronbach’s Alpha if tem eleted” values were examined, it was seen that there was no need to remove any item from the scale and all items were very reliable (Table 5). 

**Table 5:** Corrected item-total correlation and Cronbach’s alpha if item deleted.

Items	Corrected item-total correlation	Cronbach’s alpha if item deleted
1	0.697	0.848
2	0.729	0.846
3	0.487	0.864
4	0.685	0.852
5	0.738	0.845
6	0.330	0.872
7	0.739	0.844
8	0.311	0.875
9	0.457	0.866
10	0.613	0.861
11	0.583	0.858

Cronbach’s alpha = 0.869.

AID3.3. Constructand external validity

The relationships between XI-TR and ESSPRI, salivary flow rate test, OHIP-14 and OHRQoL-UK scale scores are given in Table 6. Correlations were categorized as low (r 0.10–0.49), moderate (r0.50–0.69), or high (r 0.70–1.00) [26]. There was determined to be a positive moderate to high correlation between XI-TR score and ESSPRI, OHIP-14 total and subscale scores (p< 0.05). A significant low to moderate correlation was determined between XI-TR score and salivary flow rate test, OHRQoL-UK total and subscale scores (p<0.05) (Table 6). These results showed that XI-TR is valid.

**Table 6:** Correlation between Xerostomia Inventory-XI (TR), ESSPRI, amount of saliva, OHIP-14 and OHRQOL-UK questionnaires.

		Test	Retest
ESSPRI	r	0.808**	0.826**
	p	0.000	0.000
Basal amount of saliva (Gr)	r	–0.611**	–0.644**
	p	0.000	0.000
Stimulated amount of saliva (Gr)	r	–0.674**	–0.691**
	p	0.000	0.000
OHIP-14 total	r	0.864**	0.846**
	p	0.000	0.000
OHIP-14 functional limitation	r	0.705**	0.666**
	p	0.000	0.000
OHIP-14 physical pain	r	0.729**	0.700**
	p	0.000	0.000
OHIP-14 psychological discomfort	r	0.535**	0.522**
	p	0.000	0.000
OHIP-14 physical disability	r	0.713**	0.699**
	p	0.000	0.000
OHIP-14 psychological disability	r	0.696**	0.674**
	p	0.000	0.000
OHIP-14 social handicap	r	0.675**	0.697**
	p	0.000	0.000
OHIP-14 handicap	r	0.753**	0.756**
	p	0.000	0.000
OHRQOL-UK total	r	–0.694**	–0.677**
	p	0.000	0.000
OHRQOL-UK symptom	r	–0.397**	–0.426**
	p	0.001	0.000
OHRQOL-UK physical status	r	–0.677**	–0.660**
	p	0.000	0.000
OHRQOL-UK psychological status	r	–0.505**	–0.483**
	p	0.000	0.000
OHRQOL-UK social status	r	–0.493**	–0.472**
	p	0.000	0.000

## 4. Discussion

The XI-TR questionnaire was found to be clinically valid and reliable for use in clinical evaluations, and rehabilitation interventions for patients with pSS. 

This questionnaire was originally developed to assess xerostomy in Australian elderly people [7]. Then it was applied to head and neck cancer patients who received radiotherapy [27] and to diabetic patients [28]. Later, it was applied to patients with pSS to evaluate the symptoms of sicca [3].

Since the basal and stimulated salivary flow rate test is an objective evaluation method, the OHIP-14, OHRQoL-UK and ESSPRI questionnaires, which examine the effectiveness of oral health on quality of life and disease activity, are widely accepted because of their reliability and validity, and they have been determined as the gold standard for determining construct validity [22,29,30]. Therefore, these assessment methods were used to evaluate the construct validity of XI-TR. There was determined to be a moderate to high positive correlation between the XI-TR score and ESSPRI, OHIP-14 total and subscale scores. A low to moderate negative correlation was determined between the XI-TR score and salivary flow rate test, OHRQoL-UK total and subscale scores. These results demonstrated that XI-TR is valid. In other version studies, only the salivary flow rate test has been used for validity [11,14]. Although it was reported that the salivary flow rate test has a low level of correlation with xerostomia findings, that no other outcome measures were used constituted a limited aspect of those studies. In the current study, the results were strengthened by the use of many valid Turkish questionnaires including xerostomia findings and quality of life, and the significant correlations between them.

Test/retest reliability measures the stability of answers to questions of a scale over time. Reliability indicates the accuracy and repeatability of the measurement performed. The more similar the scores of the test-retest measurements, the more reliable the questionnaire. [31]. The Cronbach alpha coefficient and the internal consistency determine the relationship between the items that make up the scale. The closer that the value is to one indicates high internal consistency and that it is reliable [32]. Cronbach alpha values in different language versions of the scale have been reported to be 0.89 and 0.87 for Spain, 0.86 for Korea and 0.90 for Portugal with pSS patients. In Germany, the Cronbach alpha value was 0.92 in patients with head and neck cancer. The internal consistency coefficient of XI-TR was calculated as 0.869 in this study, which was high and similar to the literature. It is recommended that the Cronbach alpha coefficient be above 0.8 in health-related studies [32]. Therefore, the score obtained from this study shows that the scale is quite reliable. 

The ICC varies between 0.0 and 1.0. The more similar the answers given by all individuals to the questions, the less variability in scoring and the ICC value will increase. In literature, ICC ranges have been reported as 0.590.91 for Spanish, 0.480.812 for Korean and 0.790.94 for Portuguese versions. In the current study, the ICC value was 0.853–0.994, demonstrating that XI-TR has test-retest reliability in patients with pSS. According to Fleiss (1986), if the ICCs is <0.40 it shows poor reliability, and if >0.75, it shows perfect reliability [33]. 

A strength of the current study was that not only the salivary flow rate test was used to evaluate xerostomia findings, but also Turkish questionnaires with validity and reliability in this subject. Another strength was that all of the pSS population evaluated participated in the retest. In studies applied in other countries, it can be seen that the initial number was not reached in the retest [11]. 

Xerostomia is not only seen in individuals with pSS. It can be recommended that in future studies, validity and reliability Turkish version studies are applied to other groups, such as the elderly [34], Parkinson’s disease patients [35], and those undergoing head and neck radiotherapy [36], in order to be used in other possible conditions that may cause xerostomia. 

In the light of all the results obtained in this study, XI- - -‘’ ‘’-TR scale is valid and reliable in Turkish for patients with pSS.

## Informed consent

This study was conducted in accordance with the Declaration of Helsinki. Approval for the study was granted by the Local Ethics Committee of Pamukkale University. All individuals were informed verbally and informed consent forms were signed. The code is 60116787-020/54466 (meeting dated 06.08.2019 and numbered 14).

## References

[ref1] Thomson WM 2007 Measuring change in dry-mouth symptoms over time using the Xerostomia Inventory Gerodontology 24 30 35 17302928 10.1111/j.1741-2358.2007.00137.x

[ref2] Galvez J Saiz E Lopez P Pina MF Carrillo A 2009 Diagnostic evaluation and classification criteria in Sjögren’s syndrome Joint Bone Spine 76 44 49 18829369 10.1016/j.jbspin.2008.02.017

[ref3] Cho HJ Yoo JJ Yun CY Kang EH Lee HJ 2013 The EULAR Sjogren’s syndrome patient reported index as an independent determinant of health-related quality of life in primary Sjogren’s syndrome patients: in comparison with non-Sjogren’s sicca patients Rheumatology 52 2208 2217 24023247 10.1093/rheumatology/ket270

[ref4] Aps J Joris Delanghe J Martens L 2003 Protein Composition of Saliva in Subjects Homozygous and Heterozygous for Cystic Fibrosis Caries Research 37 270 270

[ref5] Castro Sepúlveda D Cortés J Quest AFG Barrera MJ 2013 Oral Dryness in Sjögren’s Syndrome Patients Not Just a Question of Water. Autoimmunity Reviews 12 567 574 23207284 10.1016/j.autrev.2012.10.018

[ref6] Nikolov PN Gabor GI 2009 Pathogenesis of Sjogren’s syndrome Current Opinion in Rheumatology 21 465 470 19568172 10.1097/BOR.0b013e32832eba21 PMC2766246

[ref7] Thomson WM Chalmers JM Spencer AJ Williams SM 1999 The Xerostomia Inventory: a multi –item approach to measuring dry mouth Community Dental Health 16 12 17 10697349

[ref8] Pedersen AM Bardow A Nauntofte B 2005 Salivary changes and dental caries as potential oral markers of autoimmune salivary gland dysfunction in primary Sjögren’s syndrome BioMed Central Clinical Pathology 10 1472 1472 10.1186/1472-6890-5-4 PMC554998 15740617

[ref9] Vitali C Bombardieri S Jonsson R Moutsopoulos HM Alexander EL 2002 Classification criteria for Sjögren’s syndrome: a revised version of the European criteria proposed by the American-European Consensus Group Annals of the Rheumatic Diseases 61 554 558 12006334 10.1136/ard.61.6.554 PMC1754137

[ref10] Shiboski SC Shiboski CH Criswell L Baer A Challacombe S 2012 American College of Rheumatology classification criteria for Sjögren’s syndrome: a data-driven, expert consensus approach in the Sjögren’s International Collaborative Clinical Alliance cohort Arthritis Care and Research 64 475 487 22563590 10.1002/acr.21591 PMC3349440

[ref11] Lee J Koh JH Kwok SK Park SH 2016 Translation and validation of a Korean version of the Xerostomia Inventory in patients with primary Sjögren’s Syndrome Journal of Korean Medical Science 31 724 728 27134493 10.3346/jkms.2016.31.5.724 PMC4835597

[ref12] Hay EM Thomas E Pal B Hajeer A Chambers H 1998 Weak association between subjective symptoms or and objective testing for dry eyes and dry mouth: results from a population based study Annals of the Rheumatic Diseases 57 20 24 9536818 10.1136/ard.57.1.20 PMC1752470

[ref13] Serrano C Fariña MP Pérez C Fernández M Forman K 2016 Translation and validation of a Spanish version of the xerostomia inventory Gerodontology 33 506 512 25923398 10.1111/ger.12196

[ref14] da Mata AD da Silva Marques DN Freitas FM de Almeida Rato Amaral JP Trindade RT 2012 Translation, validation, and construct reliability of a Portuguese version of the Xerostomia Inventory Oral Diseases 18 293 298 22151408 10.1111/j.1601-0825.2011.01879.x

[ref15] Gkavela G Kossioni A Lyrakos G Karkazis H Volikas K. 2015 Translation and preliminary validation of the Greek version of the Xerostomia Inventory in older people European Geriatric Medicine 6 237 240

[ref16] Hohenberger R Baumann I Plinkert PK 2019 Validating the Xerostomia Inventory in a radiation-induced xerostomia population in German language Oral Diseases 25 1744 1750 31295368 10.1111/odi.13154

[ref17] Willumsen T Fjaera B Eide H 2010 Oral health-related quality of life in patients receiving home-care nursing: associations with aspects of dental status and xerostomia Gerodontology 27 251 257 19780842 10.1111/j.1741-2358.2009.00344.x

[ref18] Thomson WM van der Putten GJ de Baat C Ikebe K Matsuda K 2011 Shortening the xerostomia inventory Oral Surgery 112 322 327 10.1016/j.tripleo.2011.03.024 PMC3154566 21684773

[ref19] He S Wang J Li M. 2013 Validation of the Chinese version of the Summated Xerostomia Inventory (SXI) Quality of Life Research 22 2843 2847 23619629 10.1007/s11136-013-0420-y

[ref20] Anderson RT Aaronson NK Wilkin D. 1993 Critical review of the international assessments of health-related quality of life Quality of Life Research 2 369 395 8161975 10.1007/BF00422215

[ref21] Slade GD Spencer AJ 1994 Development and evaluation of the Oral Health Impact Profile Community Dental Health 11 3 11 8193981

[ref22] Mumcu G İnanç N Ergun G İkiz K Güneş M 2006 Oral health related quality of life is affected by disease activity in Behçet’s disease Oral Diseases 12 145 151 16476035 10.1111/j.1601-0825.2005.01173.x

[ref23] Naito M Yuasa H Nomura Y Nakayama T Hamajima N 2006 Oral health status and health related quality of life: a systematic review Journal of Oral Science 48 1 7 16617194 10.2334/josnusd.48.1

[ref24] Seror R Ravaud P Mariette X Bootsma H Theander E 2011 EULAR Sjogren’s Syndrome Patient Reported Index (ESSPRI): development of a consensus patient index for primary Sjogren’s syndrome Annals of the Rheumatic Diseases 70 968 972 21345815 10.1136/ard.2010.143743

[ref25] Haywood KL M Garratt A Jordan K Dziedzic K 2002 Dawes PT Rheumatology 41 1295 1302 12422003 10.1093/rheumatology/41.11.1295

[ref26] Fidelix T Czapkowski A Azjen S Andriolo A Neto PH 2018 Low-level laser therapy for xerostomia in primary Sjögren’s syndrome: a randomized trial Clinical Rheumatology 37 729 736 29119483 10.1007/s10067-017-3898-9

[ref27] Thomson WM Williams SM 2000 Further testing of the xerostomia inventory Oral Surgery 89 46 50 10.1016/s1079-2104(00)80013-x 10630941

[ref28] Borges BC Fulco GM Souza AJ 2010 Xerostomia and hyposaliva­tion: a preliminary report of their prevalence and associated factors in Brazilian elderly diabetic patients Oral Health & Preventive Dentistry 8 153 158 20589249

[ref29] Slade GD Spencer AJ 1994 Development and evaluation of the Oral Health Impact Profile Community Dental Health 11 3 11 8193981

[ref30] Naito M Yuasa H Nomura Y Nakayama T Hamajima N 2006 Oral health status and health related quality of life: a systematic review Journal of Oral Science 48 1 7 16617194 10.2334/josnusd.48.1

[ref31] Streiner DL Norman GR 1989 Health measurement scales. A practical guideto their development and use

[ref32] 1951 Coefficient alpha and the internal structure of tests Psychometrica 16 287 334

[ref33] Fleiss JL 1986 The design andanalysis of clinicalexperiments

[ref34] Petersen PE Yamamoto T. Improving 2005 the oral health of older people: the approach of the WHO Global Oral Health Programme Community Dentistry and Oral Epidemiology 33 81 92 15725170 10.1111/j.1600-0528.2004.00219.x

[ref35] Kaur T Uppoor A Naik D. 2016 Parkinson’s disease and periodontitis–the missing link? A review Gerodontology 33 434 438 25664991 10.1111/ger.12188

[ref36] Epstein JB van der Meji EH Lunn R 1996 Effects of compliance with fluoride gel application on caries and caries risk in patients after radiation therapy for head and neck cancer Oral Surgery 82 268 275 10.1016/s1079-2104(96)80351-9 8884824

